# Precise, contactless measurements of the surface tension of picolitre aerosol droplets†
†Electronic supplementary information (ESI) available: Parametrizations used to infer concentration, density, viscosity, and surface tension from refractive index for sodium chloride and glutaric acid; description of the semi-analytical T-matrix calculations; Fig. S1 and S2. See DOI: 10.1039/c5sc03184b



**DOI:** 10.1039/c5sc03184b

**Published:** 2015-10-05

**Authors:** Bryan R. Bzdek, Rory M. Power, Stephen H. Simpson, Jonathan P. Reid, C. Patrick Royall

**Affiliations:** a School of Chemistry , University of Bristol , Bristol , BS8 1TS , UK . Email: j.p.reid@bristol.ac.uk; b Max Planck Institute of Molecular Cell Biology and Genetics , Dresden , 01307 , Germany; c Institute of Scientific Instruments of the ASCR. v.v.i. , Krávolopolská 147 , 612 64 , Brno , Czech Republic; d H. H. Wills Physics Laboratory , University of Bristol , Bristol , BS8 1TL , UK; e Centre for Nanoscience and Quantum Information , University of Bristol , BS8 1FD , UK

## Abstract

The surface composition and surface tension of aqueous droplets can influence key aerosol characteristics and processes including the critical supersaturation required for activation to form cloud droplets in the atmosphere. Despite its fundamental importance, surface tension measurements on droplets represent a considerable challenge owing to their small volumes. In this work, we utilize holographic optical tweezers to study the damped surface oscillations of a suspended droplet (<10 μm radius) following the controlled coalescence of a pair of droplets and report the first contactless measurements of the surface tension and viscosity of droplets containing only 1–4 pL of material. An advantage of performing the measurement in aerosol is that supersaturated solute states (common in atmospheric aerosol) may be accessed. For pairs of droplets starting at their equilibrium surface composition, surface tensions and viscosities are consistent with bulk equilibrium values, indicating that droplet surfaces respond to changes in surface area on microsecond timescales and suggesting that equilibrium values can be assumed for growing atmospheric droplets. Furthermore, droplet surfaces are shown to be rapidly modified by trace species thereby altering their surface tension. This equilibration of droplet surface tension to the local environmental conditions is illustrated for unknown contaminants in laboratory air and also for droplets exposed to gas passing through a water–ethanol solution. This approach enables precise measurements of surface tension and viscosity over long time periods, properties that currently are poorly constrained.

## Abstract 2

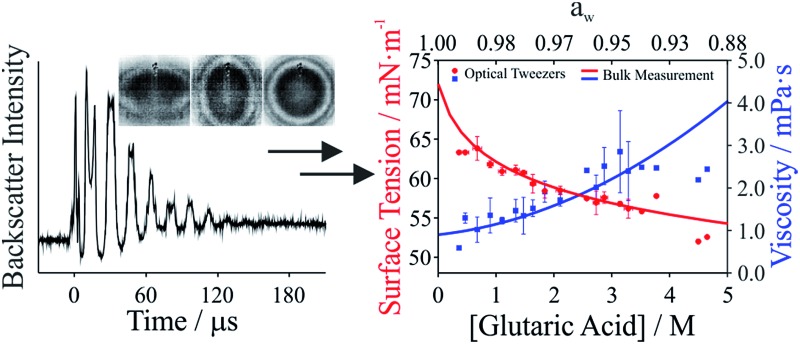
Precise measurements of the surface tension and viscosity of airborne picolitre droplets can be accomplished using holographic optical tweezers.

## Introduction

The surface tension and viscosity of droplets are crucial properties that must be understood and quantified in a range of disciplines, ranging from the evaporation and drying of sprays,^1^ the deposition of droplets on surfaces in inkjet printing,^2^ digital microfluidics/micromachinery,^3^ and cloud droplet activation in the atmosphere.^4^ Although readily probed in bulk phases, direct measurements of these properties for droplets are challenging. Reconciling bulk phase data with the high surface-to-volume ratios characteristic of dispersed droplet systems is difficult, particularly in highly dynamic systems where the transfer of volatile components across gas–liquid surfaces must be considered. Additionally, states exhibited by droplets may differ markedly from the (equilibrium) phase behaviour of bulk solutions, accessing metastable supersaturated states with solute concentrations above the solubility limit,^5^ amorphous or glassy states,^6,7^ and liquid–liquid phase separated structures.^8^ Measurements on timescales relevant to dispersed droplet systems may be similarly inaccessible through bulk measurements, as values of the surface tension and viscosity are often required for surface ages (*i.e.* the time since the surface was first generated) as short as a millisecond. Finally, conventional techniques require substantial amounts of sample, often precluding measurements on systems where the sample is limited (for example, in the collection of atmospheric aerosol samples).

In particular, the surface tension of aerosols and growing cloud droplets represents a critical uncertainty in determining the effect of aerosols on climate.^9^ Atmospheric aerosols are chemically diverse containing a myriad of organic species, and surface tension depression has been observed in aqueous aerosol extracts.^10,11^ In the absence of direct measurements, however, there has been considerable debate for many years as to the composition of particle surfaces and the magnitude of surface tensions for atmospheric aerosols and cloud droplets.^12^ The equilibrium size of an aerosol particle at a particular relative humidity (RH) is governed by an interplay between a bulk solute effect and surface curvature and is treated by Köhler theory.^13^ The former effect allows for stable particle sizes under sub-saturated RH conditions while the latter governs the magnitude of the characteristic thermodynamic barrier that must be overcome for an aerosol particle to “activate”, spontaneously growing by water condensation to form a cloud droplet. This barrier can only be overcome under supersaturated conditions with respect to gaseous water (RH > 100%), and estimating this critical supersaturation is central to predicting the fraction of aerosol particles that are activated to form cloud droplets, impacting on cloud droplet number and size, cloud albedo and persistence, and therefore radiative forcing. The fraction of the aerosol population that can act as cloud condensation nuclei (CCN) is also important for “cloud invigoration” effects, where a larger number of CCN prolong cloud development and lifetime.^14^ Some studies suggest that a surface tension equivalent to that of pure water may be appropriate for representing an activating CCN and for determining the critical super-saturation from Köhler theory.^15^ However, this assumption directly contravenes expectations for droplets containing organic solutes, the interplay between bulk and surface partitioning of organic components, and the dependence on droplet size.^16,17^ More recently, studies have suggested that organics species, which can contribute to more than half of the mass growth of nanoparticles,^18^ may reduce nanoparticle surface tension, thereby reducing the energy barrier to further growth by vapour condensation.^19^ Additionally, surface tension may play a crucial role in determining the thermodynamic state of nucleation mode aerosol, highlighting the need for reliable surface tension data for solute solutions at supersaturated concentrations.^20^

Organic surface coatings on aerosols have been observed by a number of indirect methods. Surface coatings on aerosol have been shown to play a significant role in controlling the mass transport kinetics of volatile components, such as in the transport of water across the droplet surface^20,21^ or in heterogeneous reaction rates with gas-phase species (*e.g.* N_2_O_5_ and O_3_) that must similarly pass into the bulk.^22^ Indeed, even substantial discrepancies between measurements of water condensation kinetics on droplets with fresh or aged surfaces have been attributed to contamination by organic species, which may form highly ordered surface films.^21^ Hygroscopicity studies have inferred surface tension through measurements of critical particle radii for activation and have observed surface tension depression on the order of 10% relative to water due to uptake of the small surface-active molecules methylglyoxal and acetaldehyde.^23^ Indirect inference of surface tension in this manner, however, only provides averaged values over large particle ensembles and requires solute and curvature effects to be separable. Direct and unambiguous measurements of droplet surface tensions are crucial to refine our understanding of CCN activation, the growth of cloud droplets, and the interactions that occur across gas–liquid surfaces in the atmosphere.

Two approaches have been taken to determine droplet surface tensions. One approach is the oscillating droplet method, which has been used to infer surface tension and viscosity for droplets consisting of Newtonian and non-Newtonian fluids primarily in falling droplet chains.^2,24,25^ The damped oscillatory motion of a droplet following generation can be compared with the expected natural oscillation modes of a droplet initially described by Rayleigh^26^ and extended to include viscous damping by Lamb.^27^ Using this approach, Yang *et al.* have recently shown that surface tensions inferred from measurements of the oscillation frequencies of droplets several μL down to ∼100 pL in volume over tens of ms can be reconciled with dynamic surface tension measurements made on bulk samples.^2^ Over the short timescale of these measurements, droplet composition is necessarily limited to sub-saturated solutions and the interaction with the surrounding gas-phase (crucial to determine aerosol surface properties) is minimal. Another approach adopted by Morris *et al.* uses atomic force microscopy (AFM) to probe the surfaces of aerosol particles sampled and deposited on a substrate by relating the probe tip retention force to the surface tension.^28^ This approach allows equilibrium surface tension measurements on the same droplet with varying RH (solute concentration). The benefit of this approach is that measurements are performed on individual particles deposited onto a substrate. However, this approach is necessarily more invasive as it requires contact of the AFM probe tip to the droplet, which can result in crystallization on the tip and impact the quality of the measurement.

In this work, we present contactless, non-invasive measurements of the surface tensions and viscosities of aqueous solution droplets with volumes typically 1–4 picolitres. We show that the surface tension and viscosity of a droplet can be determined with accuracies better than ±1 mN m^–1^ and ±1 × 10^–3^ Pa s, respectively, a consequence of the extremely high accuracy with which droplet size (±2 nm) and refractive index (RI, ±0.0005) are determined.^29^ The excitation and damping of oscillatory modes are observed for the smallest droplets to date (radii down to 6 μm) and approaching the fundamental hydrodynamic limit as determined by Chandrasekhar.^30^ This approach has the additional advantage that the composition is known at the moment of measurement (by virtue of the precise RI determination). Further, measurements can be made over a wide range in surface age extending from seconds to hours with the exposure of the droplet to varying gas phase conditions (for example, RH) permitting direct investigation of the dependence of surface tension and viscosity on particle composition. Performing such measurements on trapped, airborne droplets allows access to supersaturated solute states that are characteristic of aerosol. We show that surface tensions measured on picolitre droplets agree well with bulk measurements of sub-saturated solutions. Additionally, we find that droplet surfaces become contaminated on timescales of minutes unless the most stringent precautions are taken to control the purity of the local gas phase environment of the droplet. Finally, we show that the response of droplet surface tension to changes in its local gas phase environment (resulting in small changes to droplet chemical composition) can be monitored by this approach. Combined, these results describe a versatile new technique to study a crucial aerosol property that currently is poorly constrained.

## Experimental section

### Holographic optical trapping and controllable coalescence of aerosol droplets

**Fig. 1:**
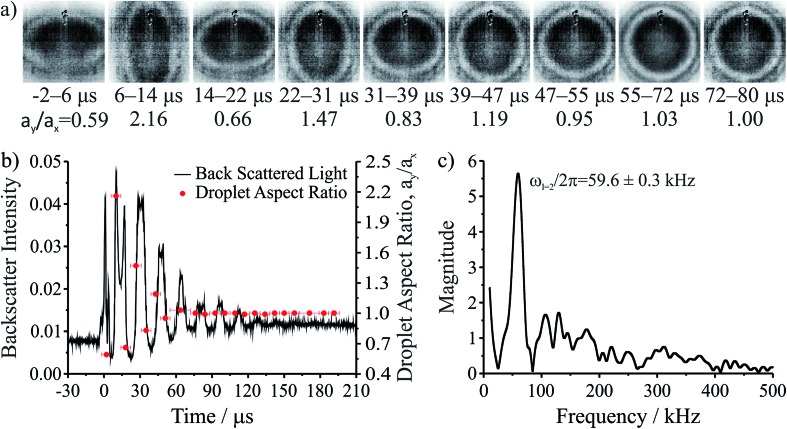
Coalescence of an aqueous ∼1 M NaCl droplet doped with the surfactant sodium dodecyl sulphate. (a) High frame rate images of the composite droplet for the first several microseconds after coalescence. Time ranges underneath each image provide the time period during which the image was taken. (b) Backscattered light collected after coalescence (left axis, time *t* = 0 μs corresponds with the moment of coalescence) and droplet aspect ratios (*a*_*y*_/*a*_*x*_) determined from the high frame rate imaging (right axis). (c) Fast Fourier transform of the backscattered light gives the frequency of the shape oscillation.

The basic optical tweezers approach used here has been described previously^5,31^ and a schematic of the apparatus is shown in Fig. S1 of the ESI.† Briefly, the optical tweezers are configured in the standard inverted microscope geometry. To form multiple steerable optical traps, the phase front of a continuous wave 532 nm laser (Laser Quantum, Opus 3W) is dynamically shaped using a liquid crystal on silicon spatial light modulator (LCOS-SLM, Hamamatsu X10468). The beam is expanded to fill the SLM display, which is conjugated to the back focal plane of a high numerical aperture microscope objective (Olympus ACH, 100×/1.25, oil) by a pair of condensing 4f telescopes. A pre-calculated sequence of kinoforms provides a simple and reproducible method by which the trapping positions can be reconfigured. Upon initiation of the user, the trap separation is varied leading to eventual droplet coalescence. The capture and relative position of trapped droplets is monitored by a camera (Dalsa Genie HM 640, CMOS). Brightfield contrast is provided by widefield illumination with a high power LED (Thorlabs, 470 nm). Similar to previous studies,^5,31^ backscattered Raman light is imaged onto the entrance slit of a 0.5 m focal length spectrograph (Princeton Instruments, Action Spectra Prop SP-2500), dispersed by a 1200 line pairs per mm grating onto a cooled CCD camera. The resulting Raman spectrum from a spherical droplet consists of a broad underlying Stokes band with superimposed resonant structure at wavelengths commensurate with whispering gallery modes (WGMs), from which the radius, RI, and dispersion can be determined with accuracies better than ±2 nm, ±0.0005 and ±3 × 10^–8^ cm respectively.^29^ The intensity of the elastically scattered component is measured using a silicon photodetector (Thorlabs, DET 110) and recorded using a low-load, high bit-rate oscilloscope (LeCroy, HDO 6034-MS). The signal is sampled with a bandwidth >100 MHz, typically >3 orders of magnitude higher than the oscillation frequency. A high frame rate camera (Vision Research, Phantom v. 7.3) is used to obtain the images shown in Fig. 1a, which have a time resolution around 8 μs. The camera acquisition is synchronized to the trigger of the oscilloscope to capture the coalescence on the camera buffer.

To isolate trapped droplets and allow environmental control, the objective focuses the beams through a coverslip (Chance Glass, #0 thickness) into a custom built trapping chamber. To populate the optical traps a fine mist of aerosol is produced using an ultrasonic nebulizer (Omron NE U22) from an aqueous solution containing involatile (or very low volatility) solutes. All chemicals are used without further purification (sodium chloride, Sigma Aldrich, 99.9999%; glutaric acid, Acros Chemicals, 99%) and dissolved in ultrapure water. The RH in the trapping cell can be controlled by varying the relative flow rates of dry and humidified nitrogen (BOC, 99.998%, 2 ppm THC max), using paired mass flow controllers (Bronkhorst), allowing fine control over the water activity of the solution droplet and control over the solute concentrations. The initial concentration of a trapped droplet has been shown to be comparable to that of the nebulized solution,^32^ which provides a coarser method for tuning the solute concentration.

### Determination of droplet composition and density

Fitting of the wavelengths of the WGMs in the droplet Raman spectra to Mie theory provides a precise determination of droplet radius and RI. RI is then related to solute concentration, droplet density, expected surface tension, and expected viscosity through different parametrizations. For NaCl, these parametrizations were obtained through polynomial fits of RI to concentration and then from concentration to density, surface tension, and viscosity across the entire range of water activity using the Aerosol Diameter Dependent Equilibrium Model (ADDEM)^33,34^ and the Aerosol Inorganics Model (E-AIM).^35^ For glutaric acid, thermodynamic data are not available, so these parametrizations were based on the relationship between measured RI (at 589 nm, Misco Digital Refractometer PA203) and solution concentration and measured RI, dispersion, and solution density (Mettler Toledo, Densito 30PX) for a range of subsaturated solutions. Parametrizations used are available in ESI.†

### Bulk tensiometry and viscometry

The surface tension of bulk aqueous solutions of sodium chloride and glutaric acid are also reported for the same solutions studied by optical tweezers. For sodium chloride, bulk measurements of surface tension allow comparison to both the optical tweezers method and calculated E-AIM results. For glutaric acid, no thermodynamic model results are available and (as discussed later) existing parametrizations of concentration to surface tension span a relatively wide range for a given solution concentration. Therefore, the surface tensions of subsaturated glutaric acid solutions were experimentally determined to more directly compare to the results of the optical tweezers approach. The surface tensions of all solutions were measured using a Wilhelmy plate tensiometer (Krüss K100).

The concentration dependence of the viscosity of glutaric acid solutions has not been reported in detail, and we report here measurements for subsaturated glutaric acid solutions measured by capillary viscometry using a Cannon-Fenske routine 200 viscometer (psl-Rheotek).

## Results and discussion

### Direct observation of oscillatory modes from coalescing droplets

The coalescence of two liquid droplets, ∼5–10 μm in radius, yields an initially dumbbell-shaped particle, which undergoes relaxation in shape *via* damped surface oscillations to a single sphere. This shape relaxation is driven by capillary forces and the minimization of surface free energy. Above some critical value of viscosity, *η*_crit_ (typically 10–20 mPa s for the droplet sizes considered here), the surface relaxation is so efficiently damped that purely aperiodic behaviour is observed. Previously we have shown that measurement of characteristic relaxation times spanning from μs to days provides a probe of the bulk properties of metastable solution droplets with viscosities spanning from 0.01 to 10^9^ Pa s, a range of 11 orders of magnitude.^5,31^ Here we consider in detail the relaxation dynamics of droplets with viscosities below *η*_crit_ within the underdamped regime, demonstrating that accurate measurements of surface tension and viscosity can be made.

Following coalescence the large initial distortion leads to the excitation of many oscillatory surface modes, with those of highest order damped over <10 μs and the longest-lived over ∼100 μs. A sequence of high frame rate images of the oscillating particle recorded with a time-resolution of 8 μs is shown in Fig. 1a. These images show a dilute sodium chloride droplet doped with the surfactant sodium dodecyl sulphate following coalescence. The composite droplet is axially situated just below the objective focal plane due to reduced buoyancy when trapping in air^36^ and laterally at the centre-of-mass of the coalescing pair. Due to the comparably weak optical forces confining the particle when compared with capillary forces,^37^ the optical forces have no influence on the relaxation dynamics and the composite particle is only drawn back into an optical trap over much longer times (>1 ms).^38,39^ Even after only a few μs, the symmetry of the oscillation appears to be consistent with only the lowest order (*l* = 2) mode for an incompressible droplet. After about 50 μs, the amplitude of the oscillation has diminished significantly. The images capture the approximate turning points in the oscillations clearly, as these are the geometries at which the sinusoidally oscillating droplet spends most time. The damping time can be readily estimated from the maxima in the droplet aspect ratio. The main uncertainty in determining the damping time from the droplet image aspect ratios arises from the camera frame rate being of similar order to the droplet oscillation frequency, so the moment of maximum shape distortion is not known with high precision relative to the oscillation period. The time-dependence of the ratio of the two droplet axes (the aspect ratio, *a*_*y*_/*a*_*x*_) is shown in Fig. 1b. The decay in the oscillation amplitude with time is evident. From the treatment of Lamb, the characteristic damping time for the *l*-th order mode (*τ*_*l*_) is related to the dynamic viscosity (*η*), radius (*a*), and density (*ρ*) of the droplet by the expression:^27^
1
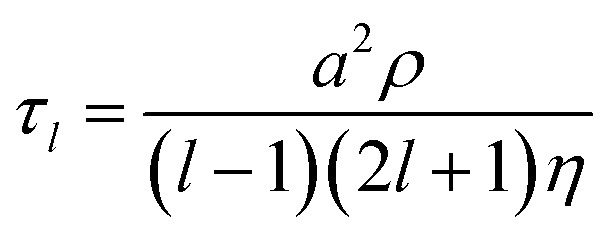



For a droplet where *a* = 10 μm, *ρ* = 1000 kg m^–3^, and *η* = 1 mPa s, the second order mode has a characteristic damping time of 20 μs, broadly consistent with the timescale of damping shown in Fig. 1b.

The natural angular oscillation frequencies (*ω*_*l*_) of the mode of order *l* can be expressed as:^26^
2
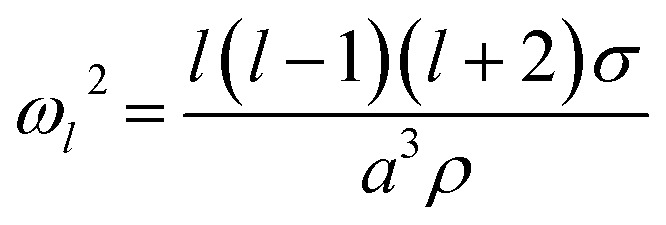
where *σ* is the surface tension. For a droplet of the size and density considered above and with *σ* = 75 mN m^–1^ the expected linear oscillation frequency (*ω*/2π) for the *l* = 2 mode is ∼100 kHz, equivalent to a period of ∼10 μs. Even with the highest frame rate accessible in the experiment, the imaging bandwidth imposes a Nyquist-limited resolution of ∼50 kHz. Therefore, a more accurate method to measure oscillation frequency and infer surface tension utilising elastic backscattered light is described below.

Fig. 1b also shows the time dependence of the light intensity backscattered from the coalescing droplet shown in the images. A correspondence is immediately apparent between the distortions in shape observed in the images (quantified by the aspect ratio) and the oscillations in backscattered intensity. A damping time can be estimated from the backscattered light by fitting an exponential to the peaks in the signal. The backscattered light measurement gives a damping time of 31 ± 3 μs. The origin of the backscattered light signal can be considered analogous to the variation in reflected intensity from a dielectric slab of variable thickness. As the path length of the light through the droplet changes with the distortion in shape, the interference of light reflected from the front and back face of the droplet leads to a modulation of the backscattered light amplitude at the modal oscillation frequencies. A Fast Fourier Transform (FFT) of the backscattered light signal allows the frequency components of the oscillation to be determined. The FFT of the backscattered light signal in Fig. 1b is shown in Fig. 1c. A single (linear) oscillation frequency corresponding to the *l* = 2 mode (*ω*_*l*=2_/2π) is evident at 59.6 ± 0.3 kHz. The linewidth of the resonance peak is a consequence of the damping rate of the oscillator. From this frequency it is possible to determine experimentally the droplet surface tension using eqn (2). However, first we validate our interpretation of the backscattered light signal using a heuristic model to demonstrate that the form of the light scattering signal and its corresponding frequency spectrum result from the geometries accessed by the oscillating droplet and the optical fields that confine it.

### Modelling the intensity of scattered light from oscillating droplets

Semi-analytical T-matrix calculations have been performed to give insight into the origin of the detected signal. Full details of the model are provided in ESI.† It is assumed that the signal derives primarily from the *l* = 2 mode and that the centre-of-mass of the composite droplet remains stationary over the course of the measurement,^38^ centred between the propagation axes of the two beams, which are separated by a distance equivalent to the radii of the initial droplets. This understanding of the position of the composite droplet relative to the trapping beams, made possible by the high frame rate images, is the key advance that enables more accurate light scatter simulations relative to our previous work.^5^ Since the optical and inertial forces are negligible, the droplet has a symmetry axis that is directed between the centres of the droplets prior to coalescence. In the model, the axis of rotational symmetry coincides with the *x*-axis, and the beams propagate along the *z* axis (out of the page). The separation between the beam axes is equivalent to the diameter of the initial droplets, *i.e.* the beams pass through the points on the *x*-axis at *y* = +(*a*/2)^1/3^ and *y* = –(*a*/2)^1/3^, where a is the radius of the combined droplet.

**Fig. 2:**
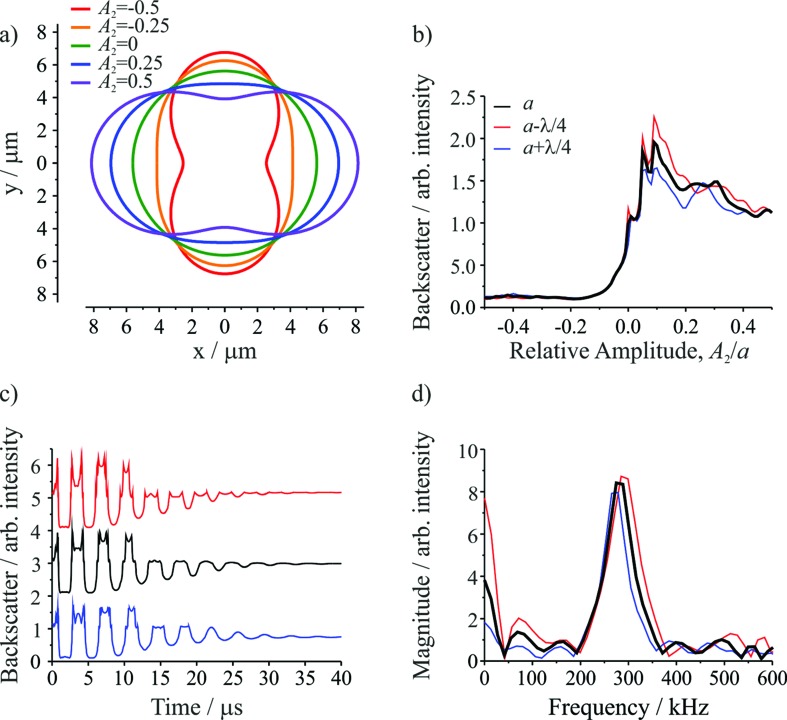
Computational modeling of droplet coalescence with the following composite droplet properties: *a* = 5.621 μm, *σ* = 74.5 mN m^–1^, *ρ* = 1059 kg m^–3^, and *η* = 1.28 mPa s. (a) Droplet shapes at various *l* = 2 distortion amplitudes (*A*_2_). The trapping beams are perpendicular to the plane of the page and pass through the points on the *x*-axis at *y* = +(*a*/2)^1/3^ and *y* = –(*a*/2)^1/3^. (b) Intensity of the integrated backscatter as a function of relative amplitude (*A*_2_/*a*). (c) Simulated time-resolved backscattered light for three droplet radii (offset for clarity). (d) Fast Fourier transform of the time-resolved backscattered light in part (c) to give the frequency of the shape oscillation. Colors in (c) and (d) follow the legend in (b).

Fig. 2a shows the droplet shape for a series of relative amplitudes, *A*_*l*_/*a*, of the *l* = 2 mode. For *A*_2_/*a* = ±0.5, the droplet shape resembles a dumbbell. For *A*_2_/*a* = 0, the droplet shape is spherical (*i.e.* the shape oscillation has no amplitude so no shape deformation occurs). For *A*_2_/*a* = ±0.25, the droplet has an elliptical shape, corresponding to an intermediate deformation between the two extremes. Fig. 2b shows the change in the integrated backscatter with variation in *A*_2_/*a* for an assumed droplet radius of 5.621 μm with *σ* = 74.5 mN m^–1^, *ρ* = 1059 kg m^–3^, and *η* = 1.28 mPa s. For comparison, integrated backscatter for two additional droplet radii are included: one where the radius is larger by *λ*/4, where *λ* is the wavelength of the trapping beam (532 nm), and another where the radius is smaller by *λ*/4. The inclusion of these two additional droplet sizes illustrates the sensitivity in the light scatter signal to relatively small (∼2%) changes in droplet radius. From this figure it is clear that at positive relative amplitudes (*A*_2_/*a* > 0) the intensity of the backscatter is much larger than the backscatter at negative relative amplitudes (*A*_2_/*a* < 0). The difference results directly from the position of the trapping beams relative to the composite droplet: when *A*_2_/*a* > 0 the composite droplet intercepts the trapping beams while it does not when *A*_2_/*a* < 0.

The time resolved signal (shown in Fig. 2c) is determined by considering the evolution of *A*_2_/*a*, governed by the damped oscillation of the surface.^5,31^ Again, the three radii were considered. The result is a backscattered light signal with additional features superimposed on an underlying oscillating signal. These features are broadly similar to those observed in the experiment (compare to Fig. 1b). The dominant influence on the signal amplitude arises from the extent to which the beams intercept the oscillating droplet surface. Higher frequency features superimposed on the underlying structure arise from the Fabry–Perot type resonance caused by optical path length changes through the droplet and the concomitant interference fringes expected in reflected light. Moreover, for the three droplet radii shown, although there are differences in the intensities of various higher frequency features, the general form of the signal is consistent, indicating that these higher frequency features should not complicate the interpretation of the experimental results.

Fig. 2d shows the corresponding FFTs of the simulated signals. Again, the dominant feature is a single resonance peak at the frequency corresponding to the *l* = 2 mode (calculated from the input parameters). Clearly, small changes in the droplet radius lead to resolvable shifts in the resonance peaks, highlighting the need for highly accurate particle size measurements. Such differences are readily resolvable in our measurements.

### Measurements of droplet viscosity and surface tension

Inferring the droplet surface tension from the modal frequencies estimated from the FFT of the elastic light scattering signal requires an accurate determination of the radius of the droplet and the density, apparent from eqn (2). We have shown previously that the radius and RI can be determined with high accuracy from the fingerprint of WGMs apparent in the Raman spectrum.^29^ Once the RI is known, the droplet composition can be inferred and the density estimated. The relationship between density, RI, and mass fraction of solute can be treated by the molar refraction mixing rule.^40,41^ We now return to consider the oscillation frequency measured for the FFT shown in Fig. 1d.

When viscous damping is significant (*η* > ∼1 × 10^–3^ Pa s for the droplet sizes considered here), the influence of this dissipation on the natural oscillation frequencies is non-negligible and the measured oscillation frequency (ω*l) is lower than that predicted using eqn (2) and is more accurately given by:^27^
3
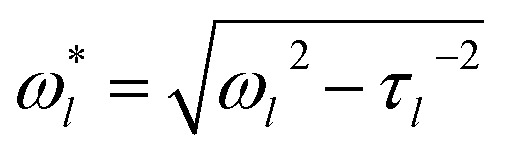



With the signal dominated by the *l* = 2 mode of oscillation, the characteristic damping time *τ*_*l*=2_ (and consequently viscosity) can determined from the exponentially decaying light scatter signal. Once *τ*_*l*=2_ is determined, the frequency and surface tension can be determined in the non-dissipative limit. While neglecting the influence of damping may be increasingly valid as the particle size increases, for the particles sizes investigated here this can lead to systematically low values for the inferred surface tension, which may be as large as 4 mN m^–1^ for the smallest, most viscous droplets studied. Note that if the viscosity is larger than the critical viscosity (*η*_crit_ ≥ 0.76 × (*aσp*)^1/2^), then surface oscillations are so efficiently damped that only aperiodic relaxation is observed, *i.e.* a slow merging of two droplets.^30^ In this limit, the surface tension cannot be obtained by this method.

**Fig. 3:**
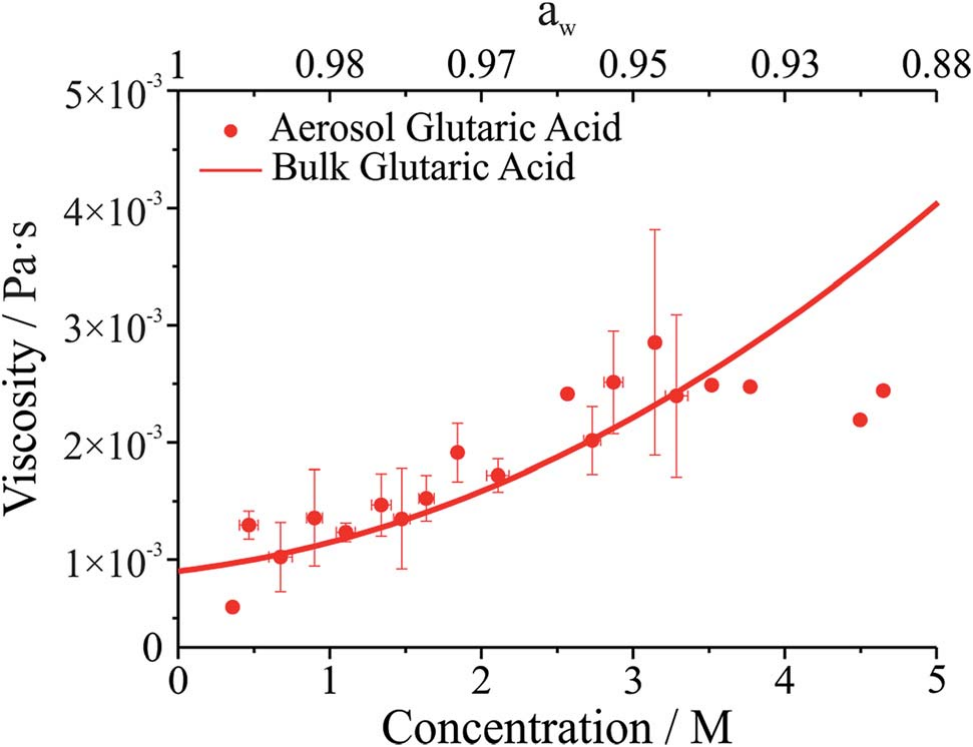
Viscosity of glutaric acid as a function of molar concentration. Symbols give measurements on droplets using the optical tweezers approach. The line corresponds to bulk capillary viscometry measurements over the same concentration range. The upper axis gives water activity (*a*_w_), which is inferred from the solute concentration.

The viscosity of aqueous organic aerosols is less well understood than for inorganic components. Numerous studies suggest that organic aerosols may exist in a highly viscous state.^6,7,42,43^ The viscosities for glutaric acid droplets inferred from the damping times are reported in Fig. 3 and compared with bulk phase measurements. To determine the viscosity, the maxima in the backscattered light signal were fit to an exponential decay to determine the damping time. Note that whereas initially the backscattered light does not follow an exponential decay, at later times it does (see Fig. 1b). Therefore, the number of maxima included in the fit was systematically varied to produce the fit most consistent with the later portion of the backscatter signal. The damping times and additional droplet parameters (*a*, *ρ*) were then used to determine *η* from eqn (1). As shown in Fig. 3, the agreement between the optical tweezers approach and bulk measurements of solution viscosity is very good.

Strictly, the linear theory of Rayleigh is valid only for small amplitude oscillations. Non-linear effects including mode coupling and frequency modulation may dominate when the distortion of the droplet exceeds 10% of the unperturbed droplet radius.^44,45^ The shape distortion is increasingly dominated by the *l* = 2 mode as the amplitude of surface oscillations decreases; the oscillation frequency then asymptotically approaches the values predicted by the linear theory. Thus, the oscillation frequency is determined in this asymptotic limit to exclude possible hydrodynamic non-linearities (see Fig. S2 in ESI†).

**Fig. 4:**
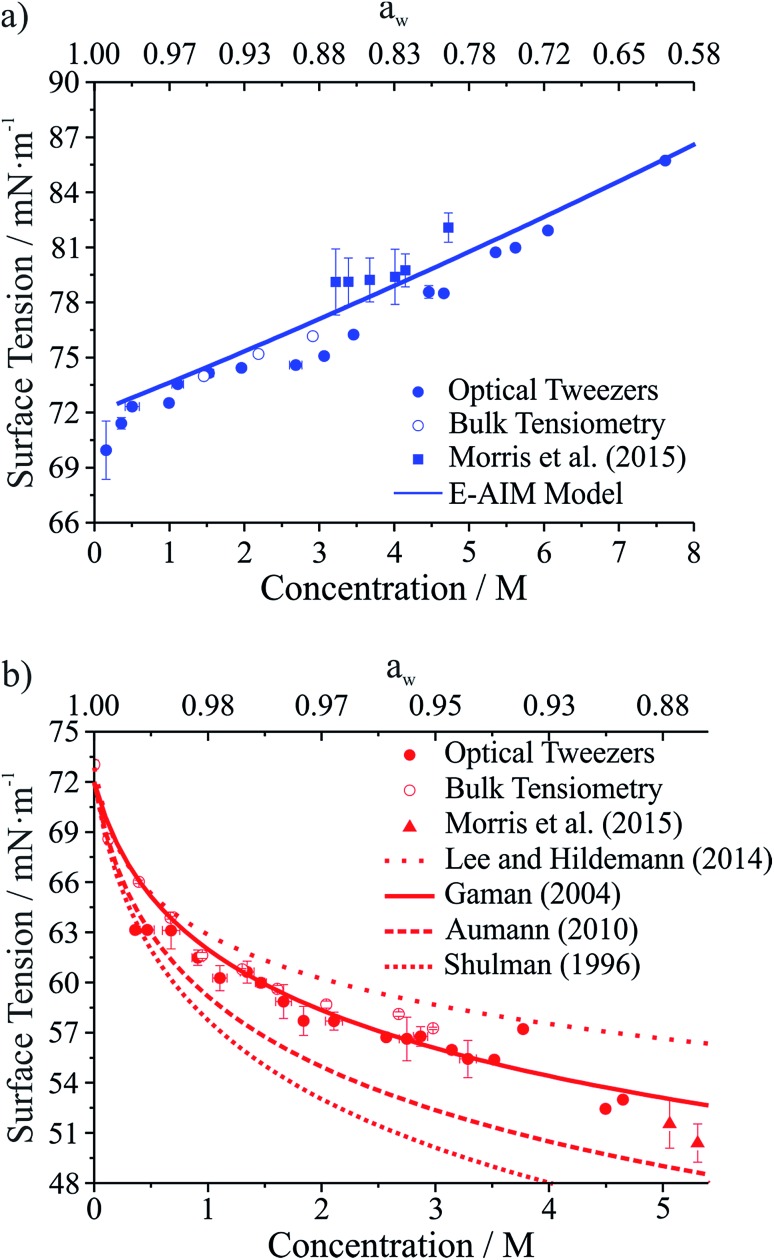
Surface tension measurements on two atmospherically relevant systems: (a) sodium chloride and (b) glutaric acid. Also included are bulk tensiometry measurements, E-AIM modeling (sodium chloride), surface tension parametrizations performed by others (glutaric acid),^47–50^ and measurements on submicron aerosol using atomic force microscopy.^28^ The upper axis gives water activity (*a*_w_), which is inferred from the solute concentration.

Droplet surface tensions inferred from the coalescence measurements are shown in Fig. 4 for droplets containing sodium chloride (Fig. 4a) or glutaric acid (Fig. 4b). Both sodium chloride and glutaric acid are atmospherically relevant compounds. Sodium chloride makes up a significant fraction of atmospheric aerosol, generated by mechanical processes at the marine surface.^46^ Similarly, the dicarboxylic acids are atmospherically prevalent and glutaric acid is often chosen as a surrogate for water soluble, surface-active organic aerosol.

In Fig. 4, the lower *x*-axis shows solute concentration, which is then used to infer water activity, *a*_w_ (shown on the upper *x*-axis). The relationship between concentration and *a*_w_ can be determined directly from E-AIM calculations for sodium chloride. For glutaric acid, *a*_w_ was determined through parametrizations of bulk concentration and density coupled with E-AIM calculations to determine the solute mass fraction as a function of RH. For the optical tweezers results, individual measurements are aggregated into 0.2 M concentration bins. Uncertainties reported on the *x*- and *y*-axes are the standard deviation of the measurements in any given bin. If no uncertainty is provided, then only one data point fell into that specific concentration bin.

In Fig. 4a, surface tensions of sodium chloride droplets obtained by the optical tweezers approach are compared with bulk tensiometry measurements and E-AIM model predictions. Additionally, results from a recent study of the surface tension of submicron aerosol by AFM are included.^28^ Overall, there is very good agreement between the optical tweezers measurements and the other measurements, validating the experimental approach. The solubility limit of NaCl is approximately 6 M, so the optical tweezers results extend into the supersaturated solute regime and appear to agree well with expected values (from E-AIM).

In Fig. 4b, surface tensions of glutaric acid droplets obtained by the optical tweezers approach are compared with bulk tensiometry measurements and a number of different parametrizations of surface tension and concentration. These parametrizations come from several independent measurements^47–51^ but we use the parameters of the Szyszkowski equation for each that are summarized in Lee and Hildemann.^49^ In addition, we show results from the AFM approach only for *a*_w_ close to the saturation limit, which is closest to our measured concentrations. Overall, our experimental results agree very well with bulk measurements. Among the various parametrizations, our measurements agree best with the parametrization of Gaman *et al.* ^48^ and appear to be consistent with the AFM results at high *a*_w_. Overall, the precision of the optical tweezers measurements typically is <1 mN m^–1^. Notably, the inorganic salt and organic dicarboxylic acid show the anticipated increase^52^ and decrease in surface tension, respectively, relative to water (72.6 mN m^–1^ at 294 K).

A related parameter encountered in surface science is the dynamic surface tension. When forming new surface area, diffusion to the interface is non-instantaneous resulting in a surface age dependent surface tension. This phenomenon is readily observed in macroscopic measurements but also in oscillating droplets. Using the falling droplet approach, Yang *et al.* found that for droplets with volumes of hundreds of picolitres and surface ages on the order of 10–100 μs, the surface tensions of droplets were considerably higher than expected based on equilibrium values.^2^ This observation is consistent with an expectation that a surfactant film (depending on surface excess and concentration) achieves an equilibrium composition on a timescale considerably longer than the surface age in such measurements, typically ∼20 ms. No such effect is seen here despite the extremely short surface ages during droplet oscillation. However, it should be noted that the relevant timescale governing the surface compositions of the two droplets is the time frame prior to coalescence when they can be assumed to reach their equilibrium values.

During the relaxation process, the surface-to-volume ratio must relax by a factor of 1.26 with initial oscillations that approach a change of this order. Over one period of oscillation in surface area (∼10 μs), a typical solute with a diffusion constant of 2 × 10^–9^ m^2^ s^–1^ may diffuse over ∼200 nm. Thus, the expansion and contraction of the surface area remains sufficiently slow that the near-surface concentration gradients established by the inorganic salt double layer and the surface excess for the organic component can rapidly compensate, maintaining a near-surface equilibrium distribution of solute molecules throughout the relaxation process. Such an observation may provide insight into the value of the surface tension during water condensation and CCN activation in the atmosphere.^53,54^ Moreover, turbulent mixing is avoided under the low Reynolds number conditions encountered here, similarly maintaining the radial concentration gradients that exist in the pre-coalescence pair.^55^ In fact, the essentially static values of surface tension measured are more comparable with those of Yamada *et al.* for coalescence under large shear deformations^25^ and Apfel *et al.* for super-deformed droplets under microgravity,^24^ where static values of the surface tension are observed for oscillating droplets. Thus, the surface tensions inferred from these measurements match the expected equilibrium values and provide a robust method for determining the surface tension of atmospherically relevant droplet compositions.

### The contamination of droplet surfaces

It has long been assumed that liquid surfaces can become contaminated through adsorption of trace impurities in the gas phase and this can have consequences, in particular, for understanding mass transfer rates across the surface of liquid droplets.^21^ Despite this presumption, direct confirmation has not been possible. Using the droplet coalescence approach it is possible to directly explore the possible time-dependent contamination of liquid surfaces. Up to this point, coalescence has been induced within 10 s of droplet capture. To study surface adsorption from the gas-phase, pairs of sodium chloride droplets are retained in the optical traps for well-defined times ranging from 10 s to >7000 s and representing more than 2 orders of magnitude in surface age.

**Fig. 5:**
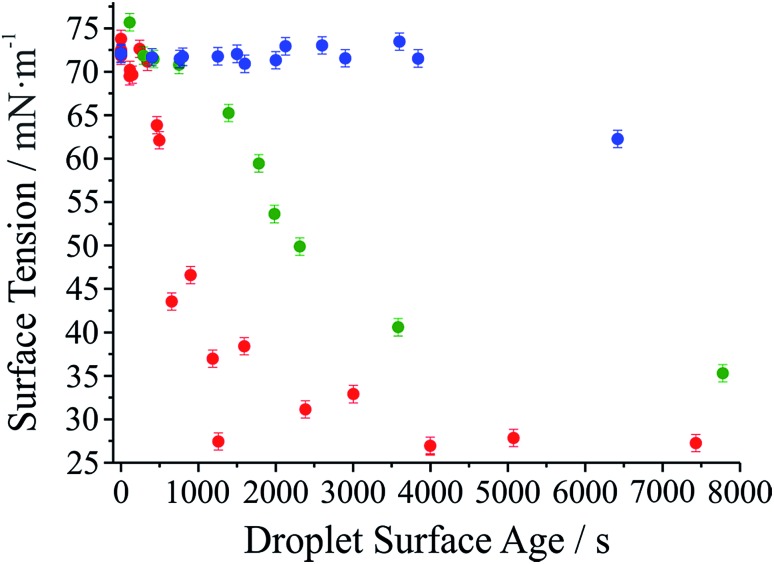
Surface tension of dilute sodium chloride droplets held in the trapping cell for varying lengths of time. Red circles correspond to measurements where no gas flow was introduced to the cell. Green circles correspond to a gas flow at 80 mL min^–1^ using a high purity N_2_ cylinder. Blue circles correspond to a gas flow at the same flow rate but using the boil-off flow from a liquid N_2_ dewar.

In Fig. 5, we report the surface tension depression recorded for variably surface aged droplet pairs. A clear decrease in surface tension is observed with surface age, converging to ∼30 mN m^–1^ when pairs of droplets were captured and held in the trapping chamber opened to laboratory air (no gas flow introduced into the cell). The time scale for the decrease in surface tension is characteristic of adsorption from the gas-phase rather than the surface partitioning of organic impurities from the droplet bulk, which would be very rapidly salted out under conditions of high ionic strength. In addition, time dependencies of two different starting salt concentrations (0.7 and 5.1 M) were found to be similar, which would not be expected if the bulk solution concentration of impurities were increased by a factor of 7.5. The bulk surface tension data recorded using a tensiometer corroborates this interpretation. It should be stressed that the level of contamination may remain very small: forming a complete monolayer would require ∼10^10^ molecules or 10^–14^ moles. Droplet surface tension relaxes to an approximately constant value around 30 mN m^–1^ after about 2000 s in the trap. This timescale for equilibration (*τ*_σ_) can be related to a Henry's law constant (*H*) for the condensing species if its molar mass (*M*) and accommodation coefficient (*α*) are known, where *a* is the droplet radius, *R* is the gas constant, and *T* is temperature:4
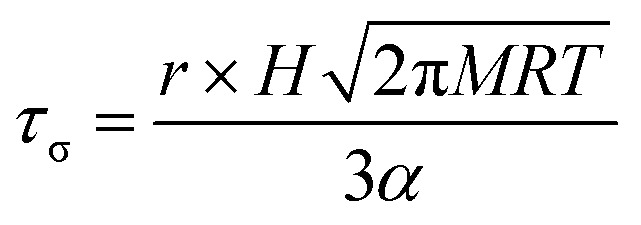



Accommodation coefficients can vary widely for different compounds but are frequently reported in the range of 0.01–1.0. Assuming a compound with molar mass of 100 g mol^–1^ condenses on an 8 μm radius droplet, the Henry's law constant would be expected to be in the range from 10^4^ to 10^6^ M atm^–1^, depending on the assumed value of *α*. Such a range for the Henry's law constant could be consistent with absorption and contamination of the droplet surface by variety of ambient species including glyoxal and oxalic acid. Absorption and dissolution would be clearly evident in the fingerprint region (500–3000 cm^–1^, 550–625 nm) of the Raman spectrum if the particle were to attain an appreciable bulk concentration of organics. Such a change is not observed here, indicating a change in composition below the Raman detection limit (typically ∼100 mM).

Further evidence that the depression in surface tension can be attributed to adsorption from the gas phase is obtained by generating droplets from a bulk solution intentionally doped with an ionic surfactant (sodium dodecyl sulphate). In each case, coalescence was induced quickly following capture (<10 s). In contrast to the data in Fig. 5, a marked depression of the inferred surface tension is immediately apparent (to 60 ± 4 mN m^–1^ over 8 individual measurements). (The range of measured surface tensions for the surfactant-doped droplets is larger than for the single component droplets due to the variability in the absolute quantity of surfactant in the droplet for each coalescence event.) Sodium chloride remains in a 100 : 1 excess and the amount of surfactant in the droplet is insufficient to form a full surface monolayer, typically containing 10^7^ to 10^8^ molecules, assuming the stoichiometry of the solute components in the droplet remains the same as in the bulk.

Also shown in Fig. 5 are the results of two other experiments where we examine the response of droplet surface tension to the environmental conditions. Initially we use a cylinder of ultrapure nitrogen, which shows only a minor improvement over laboratory air in terms of the time required for the surface tension depression. Using the boil-off from a liquid nitrogen dewar is the most reliable way of avoiding the presence of trace quantities of contaminants in the gas phase. In this case, suppression of droplet surface tension over many thousands of seconds becomes possible.

### Time-dependent observations of the adsorption of a water soluble organic and the change in surface tension

**Fig. 6:**
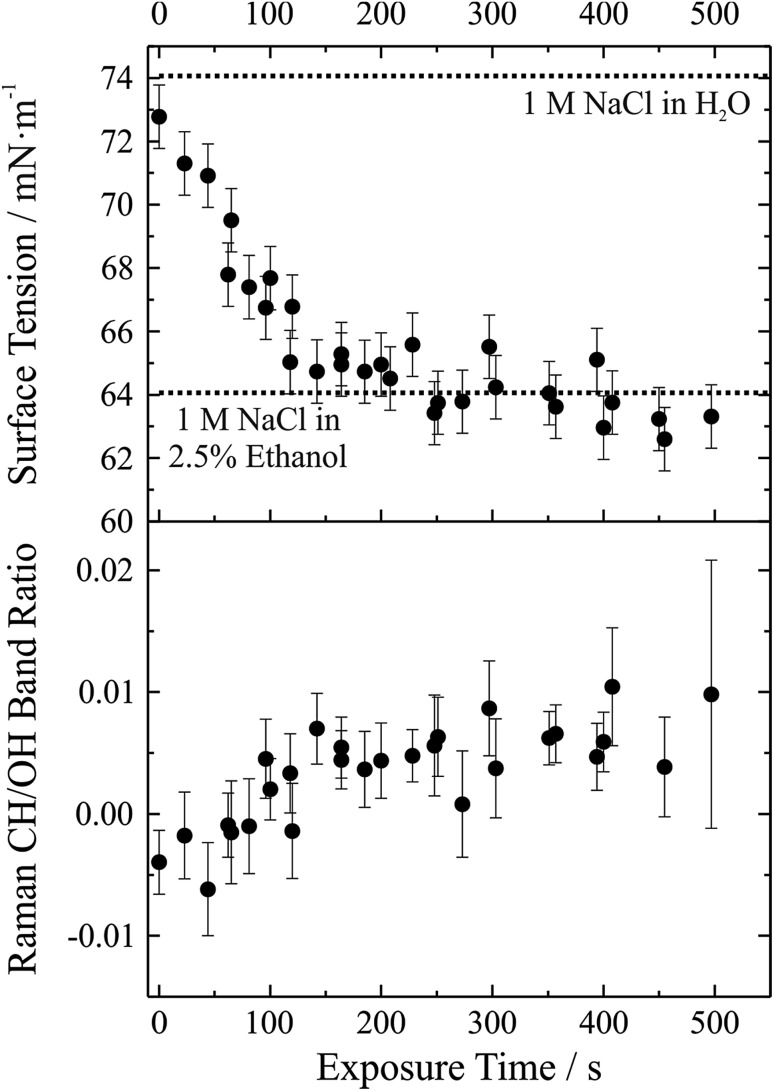
Exposure of aqueous sodium chloride to a gas flow bubbled through a 2.5% ethanol/97.5% water (volume/volume) solution. In the upper panel, symbols give measured surface tension with an assigned ±1 mN m^–1^ uncertainty. Dotted lines correspond to a 1 M aqueous NaCl solution (74 mN m^–1^) and a 1 M 2.5% ethanol solution (64 mN m^–1^). The lower panel gives the Raman CH/OH band ratio. Uncertainty bars correspond to the standard deviation of the ratio obtained from the spectra over the 20–50 seconds after coalescence.

We have demonstrated that the experimental approach is capable of performing precise measurements of droplet surface tension and that it is possible to control the local environment surrounding the trapped droplets such that undesired contamination of the droplet surface is minimized over long time periods. As a next step a gas phase “contaminant” is intentionally introduced and the response of droplet surface tension explored; that is, the response of droplet surface tension to changes in the gas phase is monitored. This process has relevance to atmospheric aerosol microphysics where aerosol chemical composition can change substantially, likely impacting surface tension. A proof-of-concept example of such an experiment is shown in Fig. 6. In this experiment, aqueous sodium chloride droplets were trapped in the cell and subjected to a vapour flow from air bubbled through a solution of water and ethanol (97.5%/2.5% volume/volume). The salt concentration in the droplets was approximately 1 M.

In the upper panel of Fig. 6, we report the change in droplet surface tension as a function of time exposed to the vapour flow. Droplet surface tension at time *t* = 0 s (*i.e.* a purely aqueous NaCl droplet) is about 73 mN m^–1^. By comparison, a surface tension of 74 mN m^–1^ was measured for a bulk 1 M aqueous NaCl solution (dotted line in the figure), in close agreement with the droplet measurements (where salt concentration is not precisely controlled but can be inferred from measurements of RI). As the droplet equilibrates to the vapour flow, the surface tension decreases and after ∼200 s stabilizes around a value of ∼63 mN m^–1^. If the liquid reservoir from which the vapour flow arises is converted into a 1 M NaCl solution (*i.e.* 1 M NaCl in 97.5% water, 2.5% ethanol by volume), bulk measurements of surface tension yield a value of 64 mN m^–1^ (dotted line in the figure), corresponding well with the droplet measurements and indicating that the droplet has equilibrated with the vapour flow.

Although the change in surface tension is substantial (∼15%), such a change only corresponds to a very minor change in droplet bulk composition. The lower panel of Fig. 6 shows the accompanying change in Raman CH/OH band ratio. The CH/OH ratio can provide a quantitative measure of ethanol uptake. The CH/OH band ratio changes on the same timescale as droplet surface tension, confirming that the change in surface tension is indeed coming from a change in the droplet composition due to absorption of ethanol. Further, the change in droplet composition is very small and barely resolvable from the change in Raman intensity. In short, this proof-of-concept experiment demonstrates that our experimental approach is capable of resolving changes in droplet surface tension for very small changes in droplet chemical composition and promises to allow us to quantitatively resolve the impact of atmospherically relevant chemistry on droplet surface tension.

## Conclusions

We have demonstrated for the first time that contactless measurements of surface tension can be made on micrometre-sized droplets containing only 1–4 pL of material. Holographic optical tweezers provide a versatile platform for conditioning aerosol, controllably inducing coalescence and probing the dynamics of the subsequent relaxation in shape *via* damped oscillations. By freeing the droplet from contact with a substrate or external probe and in concert with spectroscopic measurements of particle size and RI, this platform can be utilised as an all optical tensiometer. We demonstrated that measurements of surface tension can be made on simple organic/inorganic solute droplets with a comparable level of accuracy (±1 mN m^–1^) to bulk phase measurements. Additionally, measurements of the droplet viscosity are also possible and agree well with bulk values, essentially enabling a simultaneous determination of both surface tension and viscosity in one measurement. Therefore, this approach allows for a full exploration of the relaxation dynamics following droplet coalescence and provides complementary data to previous studies of aerosol viscosity.

Measurement of the droplet surface tension is limited to droplets with viscosities below the critical value, above which surface oscillations become overdamped (generally 10–20 mPa s for droplet radii studied here). However, droplet surface tension is key to the activation of aerosol to cloud droplets, which occurs at high water activities. As a result, activating droplets would be expected to have substantial water content and viscosities below this critical value. Droplets analyzed by this approach are typically between 5–10 μm radius, larger than the typical diameter of a CCN at activation (∼1 μm), but allowing direct measurements of comparably sized droplets for the first time. In principle, surface tension measurements over the typical range accessible to aqueous droplets are measurable by this approach.

Light scattering simulations reproduce the form of the oscillatory light scattering signal, simultaneously confirming our interpretation of the data and providing an experimental validation of models used to describe light scattering from non-spherical dielectric objects. Surface tension measurements are performed on microsecond timescales (the period during which the droplet shape is oscillating). Since these measurements agree well with bulk values, the agreement suggests that equilibrium is retained in the surface composition during shape relaxation when starting from droplets with pre-equilibrated composition. Such a result has implications for activating aerosol growing into cloud droplets from an equilibrium state, as the surface tension at any given point along the growth can be taken as its equilibrium value.

Building on the ability to measure precisely both surface tension and viscosity, we have explored the contamination of droplets due to adsorption of organics from the gas-phase, which represents a key mechanism by which droplets may obtain a surfactant coating. Recent studies have suggested the possibility of co-condensation of organic vapours during hygroscopic growth as a route to enhanced cloud droplet number concentrations.^56^ The surface tension of aqueous droplets was observed to decrease, tending towards a value for a saturated surfactant coated surface over a timescale of minutes to hours. These data have a significant implication for the assumed surface tension values of aqueous droplets in any environment and, more specifically, on the assumed value in atmospheric models of cloud droplet number. Although further studies are necessary to explore the contamination of droplet surfaces under tropospherically relevant conditions, this study suggests that assuming a surface tension of a surfactant coated surface (∼30 mN m^–1^) may be appropriate. Moreover, we show that we are able to control the local environment and monitor the response of droplet surface tension. Previous studies on aerosol extracts have shown that the organic fraction of aerosol certainly contains surfactants and a surface tension depression on the order of ∼60% could correspond to an increase in cloud droplet number concentration as large as ∼40%.^10^ Regardless, we have shown that surface partitioning and adsorption from the gas-phase can be probed in micrometre sized aerosol droplets which should provide fertile ground for further studies aiming to disentangle the various contributors to aerosol activation and the effect of aerosol on climate.
