# Supplementary material for: Precise, contactless measurements of the surface tension of picolitre aerosol droplets
Source: Chem Sci. 2015 Oct 5;7(1):274–85. doi: 10.1039/c5sc03184b (PMC5515047; doi:10.1039/c5sc03184b)
Supplement: Supplementary file 1 [file SC-007-C5SC03184B-s001.pdf]

Electronic Supplementary Material (ESI) for Chemical Science.
This journal is © The Royal Society of Chemistry 2015

Precise, Contactless Measurements of the Surface Tension of Picolitre Aerosol Droplets

Bryan R. Bzdek,[1, a] Rory M. Power,[1, 2, a] Stephen H. Simpson,[1, 3]
Jonathan P. Reid,[1, *] and C. Patrick Royall[1, 4, 5]

[1]School of Chemistry, University of Bristol, Bristol, BS8 1TS, UK
[2]Max Planck Institute of Molecular Cell Biology and Genetics, Dresden, 01307, Germany
[3]Institute of Scientific Instruments of the ASCR. v.v.i., Královopolská 147, 612 64, Brno, Czech Republic
[4]H. H. Wills Physics Laboratory, University of Bristol, Bristol, BS8 1TL, UK
[5]Centre for Nanoscience and Quantum Information, University of Bristol, BS8 1FD, UK

ELECTRONIC SUPPLEMENTARY INFORMATION

Submitted to:

Chemical Science

[a]These authors contributed equally to this work

***To whom correspondence should be addressed. Email: j.p.reid@bristol.ac.uk**

Parametrizations for Determination of Concentration and Other Properties from Refractive Index

For sodium chloride, the relationship between refractive index at 589 nm (RI) and concentration in molarity is the following: [NaCl] (M) = $-287.3\times RI^3 + 1322.3\times RI^2 - 1877.3\times RI + 833.2$. The relationship between concentration and density is: ρ_{NaCl} ($kg\cdot m^{-3}$) = $3.506\times10^{-3}\times[NaCl]^2 + 35.06\times[NaCl] + 1000.6$. The relationship between concentration and viscosity is: η_{NaCl} ($Pa\cdot s$) = $4\times10^{-5}\times[NaCl]^2 + 1\times10^{-4}\times[NaCl] + 1.1\times10^{-3}$. The relationship between concentration and surface tension is: σ_{NaCl} ($mN\cdot m^{-1}$) = $0.01\times[NaCl]^2 + 1.6\times[NaCl] + 71.8$. The data underlying these relationships were calculated using the Aerosol Diameter Dependent Equilibrium Model (ADDEM).[1, 2]

For glutaric acid (GA), parametrizations were based on experimental results from bulk, subsaturated solutions. The relationship between RI at 589 nm and concentration in molarity is: [GA] (M) = $5.8647\times RI^3 + 12.804\times RI^2 + 2.4342\times RI - 39.875$. The relationship between [GA] and density is: ρ_{GA} ($kg\cdot m^{-3}$) = $-0.4163\times[GA]^2 + 32.736\times[GA] + 997.28$. The relationship between concentration and viscosity is: η_{GA} ($Pa\cdot s$) = $9.52\times10^{-5}\times[GA] + 1.509\times10^{-4}\times[GA] + 9.017\times10^{-4}$.

Description of the Semi-Analytical T-Matrix Calculations to Simulate Light Scatter

Light scattering calculations are performed with T-matrix theory. In this approach, incident, scattered, and internal fields are centrally expanded in an orthogonal basis of spherical wavefunctions. Owing to the linearity of the optical scattering process, values for the scattered and internal field coefficients follow from the specification of the incident field coefficients and application of boundary conditions. The linearity of this operation leads to linear equations relating the incident field coefficients to the scattered or internal field coefficients. In the former case, the constant of proportionality is the T-matrix, which completely specifies the optical scattering properties of the particle. The origin and orientational frame of any particular expansion can be changed through the addition and rotation theorems for the basis functions being used. Several comprehensive accounts of this technique exist.[3, 4] In the implementation used here we use Gaussian beams of waist 4 µm, or approximately $8\lambda_0$, specified by the localized approximation.[5] The focal points of these beams are separated by the diameter of the initial droplets. Scattered field coefficients are then computed from the incident coefficients for the pair of beams, expanded about the centre of the droplet. Subsequently, the back scattered signal is evaluated by numerically integrating the far field, over the numerical aperture of the beams.

A particular problem occurs in the case of oscillating droplets. The most common method of evaluating the T-matrix of a non-spherical particle, known as the extended boundary condition method (EBCM), requires that the particle be convex (i.e. that the line segment connecting any two internal points, lies entirely within the body). The reason for this is associated with the Rayleigh hypothesis, which requires that scattered field expansions are applied only outside the smallest circumscribing sphere.[4, 6] To circumvent this problem, the T-matrix is calculated via the Generalized Point Matching Method, which constitutes a direct, point by point application of the boundary condition on the particle surface, leading to an over-specified set of linear equations, which is solved in the least square sense.[6, 7] In this case, convergence is achieved both by increasing the number of points and the number of basis functions in the expansions. In the calculations presented, spherical wavefunctions up to order 90 have been used. The cylindrical symmetry of the droplet is exploited in the calculation, so that only surface points along a longitudinal line, between the poles of the particle, need to be considered, ≈103 such points are used. This symmetry also nullifies most of the matrix elements, reducing the storage requirements.[7]

Supplementary Figures

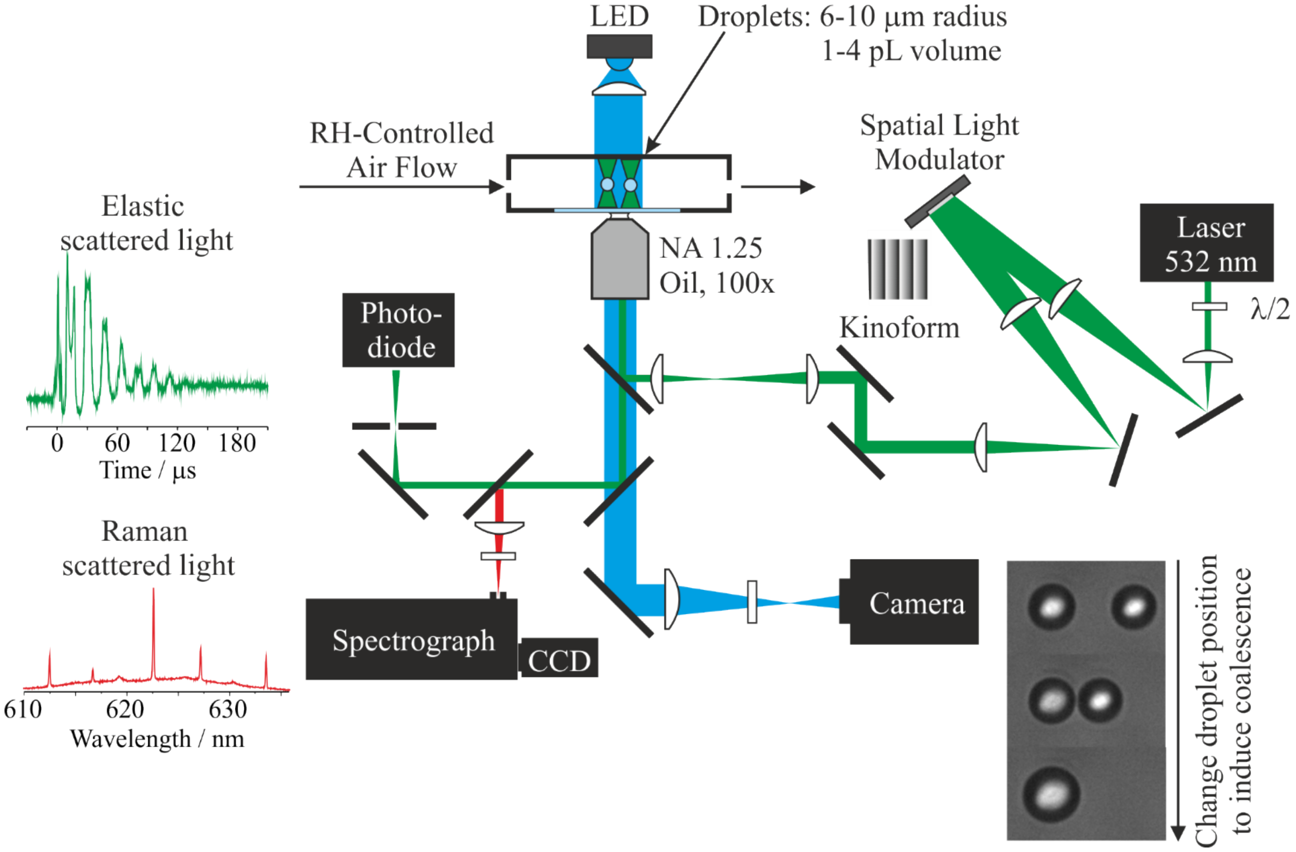

Figure S1: Schematic description of the experimental apparatus. A complete description of the experiment is provided in the main text.

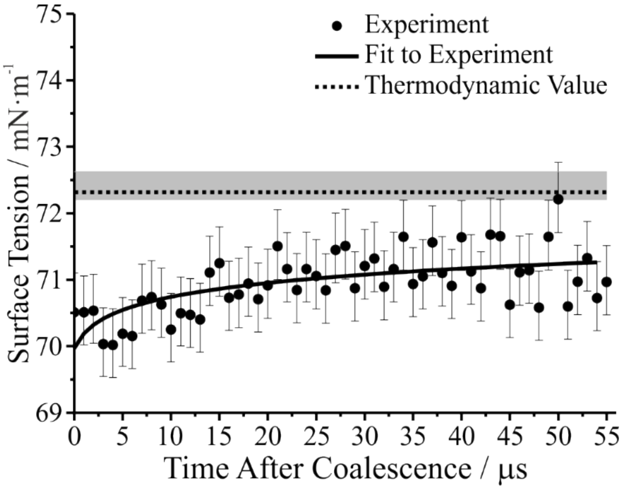

Figure S2: Dependence of the measured surface tension value on the time window over which the backscattered light signal is analysed. For example, the data point at t = 25 μs results from analysis of the backscatter signal where the backscatter signal from t = 0 to t = 25 μs is removed from the analysis. At short times after coalescence the shape distortion is very large and non-linear effects such as mode coupling and frequency modulation may impact the measured frequency. At longer times after coalescence, these effects are negligible and the measured frequency does not change. These effects are manifested in the plot by the changing surface tension value when most or all of the backscatter signal is used, compared to the relatively constant surface tension value when the first several microseconds of the backscatter trace are removed. In the analysis of the experimental data, reported surface tension values correspond to this asymptotic limit. For comparison, the expected thermodynamic value for the droplet surface tension is included as a dotted line, with the uncertainty indicated by the grey shaded region.

REFERENCES

1. D. O. Topping, G. B. McFiggans and H. Coe, *Atmos. Chem. Phys.*, 2005, **5**, 1205-1222.
2. D. O. Topping, G. B. McFiggans and H. Coe, *Atmos. Chem. Phys.*, 2005, **5**, 1223-1242.
3. M. I. Mishchenko, J. W. Hovenier and L. D. Travis, *Light Scattering by Nonspherical Particles: Theory, Measurements, and Applications*, Academic Press, San Diego, 1999.
4. M. I. Mishchenko, L. D. Travis and A. A. Lacis, *Scattering, Absorption, and Emission of Light by Small Particles*, Cambridge University Press, Cambridge, 2002.
5. G. Grehan, B. Maheu and G. Gouesbet, *Appl. Opt.*, 1986, **25**, 3539-3548.
6. F. M. Kahnert, *J. Quant. Spectrosc. Radiat. Transfer*, 2003, **79**, 775-824.
7. T. A. Nieminen, H. Rubinsztein-Dunlop and N. R. Heckenberg, *J. Quant. Spectrosc. Radiat. Transfer*, 2003, **79**, 1019-1029.
